# Emerging Intestinal Microsporidia Infection in General Population in Jiroft District, Southeastern Iran: A Cross-sectional Study in 2013–2014

**Published:** 2017-12

**Authors:** Masoomeh GHADERIPOUR, Khadijeh KHANALIHA, Mehdi MOHEBALI, Saeedeh SHOJAEE, Maryam BARKHORI, Hamed MIRJALALI, Mostafa REZAEIAN

**Affiliations:** 1. Dept. of Medical Parasitology, School of Public Health, Tehran University of Medical Sciences, Tehran, Iran; 2. Center for Research of Endemic Parasites of Iran (CREPI), Tehran University of Medical Sciences, Tehran, Iran

**Keywords:** Microsporidia, Infection, Population, Human, Iran

## Abstract

**Background:**

Microsporidia have been reported as the cause of opportunistic infections in immunocompromised patients in Iran and other countries. There is no data on prevalence of intestinal microsporidia in healthy population of Iran. This study aimed to provide preliminary data on the present status of microsporidia infection in the local healthy population in Jiroft, Kerman Province from southeastern Iran in 2013–2014.

**Methods:**

Fresh stool samples were randomly collected from 418 residents in rural 209 (50%) and urban 209 (50%) areas of Jiroft. All of the collected samples were concentrated with conventional formalin-ether, stained with Ryan blue. Microscopic examination was performed with high magnification on each sample separately for the demonstration of microsporidia spores.

**Results:**

Microsporidial spores were identified in 41 out of 418 (9.8%) samples including 16.41(39%) from rural areas and 25.41(61%) from urban areas. In general, there was no significant difference between sex, age, job, education, and contact with soil and livestock, water supply, gastrointestinal disorders and microsporidia infection among general population in Jiroft.

**Conclusion:**

*Intestinal microsporidia infection without clinical manifestations is* prevalent in general population resident in southeastern Iran. Appropriate molecular methods are needed for microsporidia species identification.

## Introduction

Microsporidia become known as one of responsive agents of opportunistic infections in patients with AIDS and other immunocompromised individuals (1). Microsporidian infections of human sometimes could cause a disease called microsporidiosis. Up to now, 8 genera and 14 species of microsporidia have known as causative agent of human infection (2). Microsporidia have the smallest known eukaryotic genomes that characterize all known species (3). Microsporidial genera such as Enterocytozoon**,** *Encephalitozoon species including Encephalitozoon cuniculi*, *E. hellem*, and *E. intestinalis* are the most common microsporidia associated with human disease (4). Although microsporidiosis has been identified in a broader range of human populations including travelers, children, the elderly, organ transplant recipients, patients with malignant disease and diabetes (5), Immunocompromised patient, especially HIV-positive population is considered more susceptible to parasite infections than other populations (6).

The most common clinical symptoms among immunodeficient patients are chronic diarrhea, weight loss, systemic diseases and in some cases mortality, While *immunocompetent* persons often have mild or self-limiting disease (1,7). Microsporidian diarrhea has been reported in travelers because of an outbreak related with contaminated cucumbers (5). The microsporidian infections have been occurred using contaminated water supplies by wild and domestic animal or food that produced from farm animals, shows importance of this food and water zoonotic disease (7).

The use of antiretroviral therapy for preventing opportunistic infection in HIV positive patient has decreased the rate of microsporidian infection, however, is more reported as self-limited diarrhea and other intestinal complications in non-HIV immunodeficient individuals and in healthy people (8).

Asymptomatic microsporidiosis have been reported among immunocompetent people and seroprevalence data showed that human in exposes with infection (1,9–11). Diagnosis of microsporidia is according to identification of spores by parasitological methods like *chromotrope* 2R, *Calcofluor* white (12) and molecular method (11,13) described before. Enterocytozoon bieneusi *has been reported as an* important cause of chronic diarrhea in persons infected with human immunodeficiency virus (HIV) in Iran and other countries (1, 13–15). Enterocytozoon *intestinalis* is also another cause of chronic diarrhea in HIV+ population and *immunocompromised* people (6, 16). *Encephalitozoon cuniculi* is a microsporidia species most commonly recognized as a cause of renal, respiratory, and central nervous system infections in immunosuppressed patients (4).

Wildlife species like foxes could be significant reservoirs of *E. cuniculi* infection for both domestic animals and humans (17), and findings of the *E. cuniculi* from human and rabbits from different regions of the China revealed zoonotic sources of infection involving rabbits to be important route of transmission in humans (18).

Recently researchers have been focused on emerging opportunistic infection in Iran. Some studies have been described the frequency of intestinal microsporidia and *coccidian* parasites in HIV positive patient [13, 19, 20] and liver transplant population from Iran (21). However, there is no data about prevalence of intestinal microsporidia in general population in Iran.

Regarding increasing trend of *immunocompromised patients like* HIV+ patients and transplant recipients and cancer patients, identification of the microsporidia in clinical samples is important to treat and reduce dissemination risks among patients in Iran.

This study aimed to provide preliminary information on the prevalence of microsporidia in the local general population in Jiroft, Kerman Province in Iran in 2013–2014.

## Materials and Methods

### Study area

**Fig. 1:**
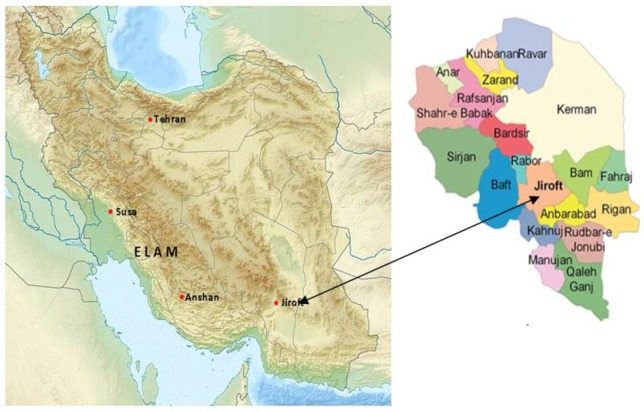
Situation of Jiroft in Kerman Province in Iran

This study was performed in Jiroft, south Kerman Province, and southern Iran (Fig. 1).

It is comprised of four districts, fourteen rural districts, and four towns*. Jiroft* has a population of 277748, among whom 121988 (36%) lived in urban areas while the rest resided in villages. The town of Jiroft is located approximately 248 km^2^ southeast of Kerman. (https://en.wikipedia.org/wiki/Jiroft_County).

### Samples Collection

In this cross-sectional study, 418 individuals including residents of rural and urban areas were *enrolled* to evaluate intestinal microsporidia infections during 2013–2014. Sample collection was done according to cluster random sampling. Information about the infection was collected by filling up questionnaire by people in the community. we designed some simple questions about the basic amenities available, the use of sanitary toilets, water supply including pipe, spring or well-drinking water, gender, age, education, job, gastrointestinal disorder and contact with soil and animals that may be source of infection.

Fresh stool samples were collected from all individuals and examined by formalin-ether concentration. All of samples were examined for intestinal microsporidia infections in the department of Medical Parasitology, School of Public Health, Tehran University of Medical Sciences.

Informed consent was taken from the participants and the study was approved by Ethics Committee of Tehran University of Medical Sciences, Iran.

### Ryan blue staining method

Conventional formalin-ether was done for all samples, for detection of intestinal microsporidia parasites, thin slides were prepared from stool samples and after methanol fixation allow the slides to dry for 10 min and all of slides were stained according to Ryan blue staining method (22). Finally, slides were examined using light microscope with 1000-x magnification.

The analysis was performed using SPSS ver. 18 (Chicago, IL, USA) and Chi-square.

## Results

**Fig. 2:**
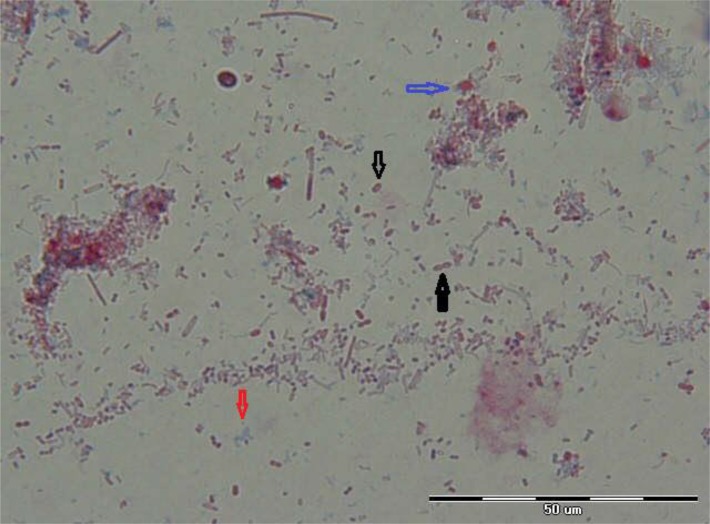
Appearance of microsporidial spores in aniline blue staining method; black arrow: microsporidial spore; blue arrow: fungal elements; red arrow: bacterial arrow (1000 magnification, original picture)

Overall 418 individuals were enrolled in this study; 229 (54.8%) were female and 189 (45.2%) were male and 209(50%) lived in rural and 209(50%) lived in urban areas of Jiroft. Transparent ovoid microsporidian spores were observed measuring 1–1.5 μm size with a background of blue staining fecal bacteria in positive stool samples with 1000 magnification (Fig. 2).

Microsporidial spores were identified in 41 out of 418(9.8%) samples, including: 20.41 (48.7%) female and 21.41 (51.2%) male.

In general from 41 microsporidia positive samples 16.209(7.7%) samples were from rural areas that 7(43.8%) were male and 9(56.2%) samples were female and 25.209(12%) samples associated with urban areas including, 14(56%) male and 11(44%) female samples.

The results of microsporidia infections inhabitant in rural and urban areas in different age groups are summarized in (Table 1). The most infected intestinal microsporidia 12(29.3%) related to children aged 0–9 yr old.

**Table 1:** Microsporidial infection rate according to rural and urban areas and age among 418 general populations in Jiroft, Southeastern Iran in 2013–2014

	***Rural areas***		***Urban areas***		***Total***	
	**Positive infection**	**Negative infection**	**Positive infection**	**Negative infection**	**Positive infection**	**Negative infection**
Age (yr)	Number (%)	Number (%)	Number (%)	Number (%)	Number (%)	Number (%)
≤9	7(43.8)	60(31.1)	5(20)	42(22.8)	12(29.3)	102(27.1)
10–19	4(25)	33(17)	3(12)	35(19.1)	7(17.1)	68(18.0)
20–29	1(6.2)	32(16.6)	5(20)	40(21.7)	6((14.6)	72(19.1)
30–39	2(12.5)	32(16.6)	5(20)	29(15.8)	7(17.1)	61(16.2)
40–49	0(0)	15(7.8)	2(8)	21(11.4)	2(4.9)	36(9.5)
≥50	2( 12.5)	21(10.9)	5(20)	17(9.2)	7(17.1)	38(10.1)
Total	16(100)	193(100)	25(100)	184(100)	41(100)	377(100)

The prevalence of intestinal microsporidia between housewives 10 (24.4%) and students 10 (24.4%) was more than other people.

Although the infected people who had contact with soil in rural 11(68.8%) and urban areas14 (56%) were more than people who had not any contact with soil in this mentioned areas 5(31.2%) and 11(44%) respectively, there was no significant differences between infection and contact with soil in current study.

Frequency of intestinal microsporidia in people who had no contact with livestock in rural and urban areas 27(65.9%) was more than people who had contact with livestock 14(34.1%).

In this study, 37/41(90.2%) of infected people were able to access to piped water and 4.41 (9.8%) of them used untreated water.

Although infection rate of microsporidial infection was more in people who had abdominal pain in this study than people without gastrointestinal discomfort, there was no statistical significance between gastrointestinal discomfort and microsporidia infection.

In general, there was no significant difference between microsporidia infection and sex, age, job, education, contact with soil and livestock, water supply and gastrointestinal discomfort in this study.

## Discussion

The prevalence of microsporidia infection was between 0%–50% in different areas using parasitological and molecular diagnostic method (23). Prevalence of microsporidia has also been reported between 7 %–50% in patients with AIDS (24).

There are few reports on microsporidian infection in HIV+ population and transplant recipient from Iran. In a study, 356 HIV+ patients were evaluated in Shiraz, Iran, eight with persistent chronic diarrhea were found to be positive for *E. bieneusi* (genotypes D and K) using microscopic examination and the nested PCR technique (13) and in another study *E. bieneusi* (genotype D) were detected in 6.81% of liver transplant children (21).

In a study, 25 out of 81(30.86%) stool samples from HIV+/AIDS patients from Tehran were positive for intestinal *E. bieneusi* infection, by nested PCR and staining method. Although chronic watery or moderate diarrhea was existed in 13 (52%) of positive cases no statistically significant difference was found (19).

However, in a few cases, infections in immuno-competent people also have been observed (25–27). The actual frequency of intestinal microsporidial infections in healthy people is unknown. Microsporidia still are, often overlooked and misdiagnosed because they are not specifically identified in most diagnostic labs, they are rather small, needed different staining methods and expert technologist (1,5).

Regarding improved diagnostic methods, microsporidia have become more described even in healthy people without any clinical manifestation (27–29).

Present study is the first report on microsporidian infection in healthy people in Jiroft, south of Iran. In our study, prevalence of microsporidial infection was 9.8% in rural and urban areas by parasitological method. There was no significant relationship between infection and gastrointestinal disorders. The most infected intestinal microsporidia 12.41(29.3%) belonged to children aged 0–9 yr old but there was no significant difference (*P*-value: 0.7) between age and microsporidian infection in this study. In Spain, 17% of 60 HIV-negative patients were infected with microsporidia (26).

The frequency of microsporidiosis in healthy people in Cameroon was noticeably high. In a study, 67.5% of the 126 healthy people were positive for microsporidiosis by calcofluor white and randomly selected samples were confirmed *E. bieneusi* (27). The frequency of *E. bieneusi* was 35.7%, in patient with HIV positive and tuberculosis coinfection, but this rate was only 24.0% among only tuberculosis patients. The infection rate among healthy people was significantly higher from those of both groups of tuberculosis positive patients. The most infection rate was among juvenile group, and the most infection degree (2.5) was in children (27).

Another report of such a high prevalence of microsporidian infections is related to Ugandan children with persistent diarrhea, of which 32.9% were shedding of *E. bieneusi* (28).

Prevalence of *E. bieneusi* infections among children aged between 3–36 months with diarrhea was17.4%, however, this rate was 16.8% in control group of children in Uganda and the highest infection was found in rainy seasons. There was no significant difference between *E. bieneusi* infection and low growth or low weight infants and acute diarrhea (29).

In our study, the highest infection rate was found among children aged between 0–9 yr old. A high prevalence of microsporidia was found in immunocompetent people in a study. The frequency of microsporidian infection in patients with acute diarrhea and chronic diarrhea were 27.0% and 34.1% respectively, surprising this rate of infection in health people was 45.5%. The higher rate of microsporidia infection was found in soft stools (51.4%), and the rate of infection increased with increasing of age. There was no statistical difference between microsporidia infection and the presence of clinical presentation (11).

In another study, 22 out of 72 (30.6%) stool samples from healthy Roma, Slovak children were positive by Rylux D, staining and Real-Time PCR. *E. bieneusi* (genotype A) was detected in 3 (4.2%) samples and *E. cuniculi* (genotype I) in 19 (26.4%) samples (30).

Some studies demonstrated relation between microsporidian infection and clinical manifestation like diarrhea (31); however, there was no relation between infection and diarrhea in healthy people and HIV+ patients in other studies (10, 26). There was no significant relationship between infection with microsporidia infection and diarrhea in our study too.

In general, prevalence of microsporidial infection was 9.8% in rural and urban areas in Jiroft by parasitological method and there was no significant difference between sex, age, job, education, and contact with soil and livestock, water supply, gastrointestinal discomfort and microsporidial infection in this study.

Regarding sources of human microsporidial infections and modes of transmission are still unknown, further accompanied studies are required to investigate microsporidia infection in different parts of Iran. Additional studies of immunocompetent people in other parts of Iran, as well as in other countries by molecular and parasitological methods, are needed for better understanding of *microsporidiosis* epidemiology.

## Conclusion

*Microsporidial* infections in general population of southeast of Iran is prevalent. *No signs and symptoms* are observed in general population resident in the infected individuals. Appropriate molecular methods are needed for identification of microsporidia species.

## Ethical considerations

The study was performed in compliance with current national laws and regulations. Ethical issues (Including plagiarism, informed consent, misconduct, data fabrication and/or falsification, double publication and/or submission, redundancy, etc.) have been completely observed by the authors.
